# Contemporary Insights Into the Genetics of Hypertrophic Cardiomyopathy: Toward a New Era in Clinical Testing?

**DOI:** 10.1161/JAHA.119.015473

**Published:** 2020-04-18

**Authors:** Francesco Mazzarotto, Iacopo Olivotto, Beatrice Boschi, Francesca Girolami, Corrado Poggesi, Paul J. R. Barton, Roddy Walsh

**Affiliations:** ^1^ Cardiomyopathy Unit Careggi University Hospital Florence Italy; ^2^ Cardiovascular Research Center Royal Brompton and Harefield NHS Foundation Trust London United Kingdom; ^3^ National Heart and Lung Institute Imperial College London United Kingdom; ^4^ Department of Clinical and Experimental Medicine University of Florence Italy; ^5^ Genetic Unit Careggi University Hospital Florence Italy; ^6^ Department of Paediatric Cardiology Meyer Children's Hospital Florence Italy; ^7^ Department of Clinical and Experimental Cardiology Heart Center Academic Medical Center Amsterdam the Netherlands

**Keywords:** genetic association, genetic testing, hypertrophic cardiomyopathy, Genetic, Association Studies, Cardiomyopathy, Hypertrophy, Diagnostic Testing

## Abstract

Genetic testing for hypertrophic cardiomyopathy (HCM) is an established clinical technique, supported by 30 years of research into its genetic etiology. Although pathogenic variants are often detected in patients and used to identify at‐risk relatives, the effectiveness of genetic testing has been hampered by ambiguous genetic associations (yielding uncertain and potentially false‐positive results), difficulties in classifying variants, and uncertainty about genotype‐negative patients. Recent case‐control studies on rare variation, improved data sharing, and meta‐analysis of case cohorts contributed to new insights into the genetic basis of HCM. In particular, although research into new genes and mechanisms remains essential, reassessment of Mendelian genetic associations in HCM argues that current clinical genetic testing should be limited to a small number of validated disease genes that yield informative and interpretable results. Accurate and consistent variant interpretation has benefited from new standardized variant interpretation guidelines and innovative approaches to improve classification. Most cases lacking a pathogenic variant are now believed to indicate non‐Mendelian HCM, with more benign prognosis and minimal risk to relatives. Here, we discuss recent advances in the genetics of HCM and their application to clinical genetic testing together with practical issues regarding implementation. Although this review focuses on HCM, many of the issues discussed are also relevant to other inherited cardiac diseases.

**Nonstandard Abbreviations and Acronyms**
ACMG: American College of Medical Genetics
AMP: Association of Molecular Pathologists
CNV: copy number variant
DCM: dilated cardiomyopathy
DEff: diagnostic effectiveness
ESP: National Heart, Lung and Blood Institute Exome Sequencing Project
ExAC: Exome Aggregation Consortium
GWAS: genome‐wide association study
HCM: hypertrophic cardiomyopathy
LMM: Partners Healthcare Laboratory for Molecular Medicine
LV: left ventricular
LVOTO: left‐ventricular outflow tract obstruction
LVWT: left‐ventricular wall thickness
NCBI: National Center for Biotechnology Information
NGS: next‐generation sequencing
SCD: sudden cardiac death
VUS: variant of uncertain significance
WES: whole‐exome sequencing
WGS: whole‐genome sequencing

Hypertrophic cardiomyopathy (HCM) is an inherited disease of cardiac muscle characterized by substantial heterogeneity in morphology, clinical manifestation, genetic etiology, and outcome.^1^ HCM is diagnosed in the presence of a maximum left‐ventricular wall thickness of at least 15 mm when this is not solely explained by abnormal loading conditions (such as hypertension and aortic stenosis), with other typical features including myocyte disarray and interstitial fibrosis.^2^ Although HCM is considered a Mendelian disease with largely autosomal dominant inheritance, incomplete penetrance and variable expressivity are commonly observed in affected families.

As well as being a relatively common (estimated to affect 1 in 500 people)^2^, ^3^, ^4^ and medically important condition, HCM has been an important model disease for studying issues that are shared among many inherited cardiac diseases. Research into the genetic basis of HCM has been ongoing for almost 30 years and early implementation of clinical genetic testing has yielded extensive data sets for use in research studies. However, several issues have limited the impact of HCM genetic testing in clinical practice:

1. Over half of patients tested have a negative genetic test result (ie, no definitive or putative pathogenic variant is detected).
2. The significance of many detected variants has been difficult to ascertain.
3. The limited correlation between genotype and clinical phenotype has constrained the use of genetic knowledge in directing clinical management.
4. The ever‐increasing number of genes apparently associated with disease has clouded our understanding of the genetic architecture of HCM.

Despite these open challenges, recent findings have transformed our understanding of the genetic basis of HCM and offered the potential for more accurate, sensitive, and impactful application of genetic testing. These findings have been driven by the development of population genetic databases that have revolutionized our understanding of rare genetic variation in health and disease and by the accumulation of large patient cohorts and disease registries. Collaborative models of data sharing, together with the development of consensus guidelines for variant interpretation and validation of disease‐gene associations, have proven invaluable for improving our knowledge of rare genetic diseases.

Here, we discuss the achievements earned in understanding the genetics of HCM in the past 30 years, the clinical implications of the current reassessment of genetic associations, the need to manage the genotype‐negative HCM patient in a coherent and effective manner, and the challenges that need to be addressed before we can adopt a patient‐specific, individualized approach in HCM. While focusing on HCM, the issues discussed are also broadly relevant to our understanding of other cardiomyopathies and inherited cardiac diseases.

## Genetic Associations

HCM was first recognized over 50 years ago as a familial myocardial disease with increased risk for sudden death, variable disease expressivity, and natural history.^5^, ^6^ After more than 2 decades during which the cause of HCM remained unknown, the dawn of a molecular era arrived in 1989, when the first genetic locus (14q1) associated with the condition was detected by means of a linkage analysis on a large kindred group comprising 96 individuals and spanning 4 generations.^7^ Shortly after, the associated genomic region was narrowed further by additional linkage studies on the same family, restricting the region containing the genetic defect to locus 14q11‐12,^8^ and consequently defining a potential causal role for one of the two highly homologous genes encoding myosin heavy chains: *MYH6* and *MYH7*. The responsible gene was identified the same year as *MYH7*, with the identification of the causative missense substitution p.Arg403Glu.^9^ The same month, another study from the same research group was published to demonstrate the genetic heterogeneity of HCM, as co‐segregation of variants at the *MYH7* locus with HCM was observed in only 2 of 4 affected pedigrees, highlighting the existence of alternative genetic causes at other loci.^10^

After these pivotal discoveries, the genetic repertoire of HCM was gradually expanded throughout the decade to include seven additional sarcomeric genes (*MYBPC3, TNNT2, TPM1, MYL2, MYL3, TNNI3, ACTC1*)^7^, ^8^, ^9^, ^10^, ^11^, ^12^, ^13^, ^14^, ^15^, ^16^, ^17^, ^18^, ^19^ (Table 1), incontrovertibly associated with HCM by studies based on linkage analyses in large and/or multiple affected families and functional experiments. We refer to these eight “core” disease genes with the term “validated genes” in this review. Simultaneously, definitive genetic associations with genocopies of HCM (ie, metabolic diseases characterized by a phenotype mimicking HCM but caused by genetic variants in different genes) were demonstrated, starting with Pompe disease^20^, ^21^, ^22^, ^23^, ^24^, ^25^, ^26^, ^27^, ^28^, ^29^, ^30^, ^31^, ^32^, ^33^ (Table 2). Besides Pompe disease, Fabry disease, PRKAG2‐cardiomyopathy, Danon disease, and TTR‐amyloidosis, other conditions (due to mitochondrial dysfunctions) can seldom present resembling isolated HCM, such as Friedreich's ataxia and Leigh syndrome, caused by variants in the *FXN* and *COX15* genes, respectively.

**Table 1:** Breakdown of the Original Linkage Studies Demonstrating the Co‐Segregation of Genetic Variants and Hypertrophic Cardiomyopathy in Large Pedigrees and Incontrovertibly Associating Sarcomeric Genes With HCM

Gene	Protein	Demonstrated Associations	Year	Reference No.	Inheritance	N Pedigrees—(Total Size)	Max LOD	Notes
MYH7	Beta‐myosin heavy chain	Locus 14q1	1989	7	AD	1 (96)	9.37	···
		Locus 14q11‐12	1990	8		1 (96)	4.62	···
		Gene	1990	9		1 (96)	15.9	···
		Genetic heterogeneity of HCM	1990	10		4 (173)	10.85	···
TNNT2	Cardiac troponin T	Locus 1q3	1993	11	AD	3 (97)	8.47	···
		Gene	1994	12		1 (70)	6.3	···
MYBPC3	Myosin‐binding protein C	Locus 11p13‐q13	1993	13	AD	1 (54)	4.98	···
		Gene	1995	14		2 (46)	3.74	···
TPM1	Alpha tropomyosin	Locus 15q2	1993	15	AD	2 (87)	6.02	···
		Gene	1994	12		2 (87)	6.94	···
MYL3	Essential myosin light chain 3	Gene	1996	16	AD	1 (53)	6.2	···
TNNI3	Cardiac troponin I	Gene	1997	17	AD	1 (18)	3.1	···
MYL2	Regulatory myosin light chain 2	Gene	1998	18	AD	3 (47)	2.41 (estimated)	a
ACTC1	Alpha actin (cardiac muscle) 1	Gene	1999	19	AD	1 (22)	3.6	···

AD indicates autosomal dominant; and HCM, hypertrophic cardiomyopathy.

a Although an LOD score of 2.41 is below the universally accepted threshold of LOD=3 for co‐segregation to be considered unequivocal, in the years following this original association with HCM, further evidence about the gene's disease‐causing role in HCM gradually accumulated^20^, ^21^, ^22^, and collectively made the association incontrovertible. A larger family with HCM due to a pathogenic variant in MYL2 was reported recently, with a LOD score for co‐segregation of 4.51.^23^

**Table 2:** List of the Genetic Associations With Metabolic/Infiltrative Genocopies of HCM, Originally Demonstrated Through the Collation of Different Types of Evidence (See References and Notes), Alongside the Main Currently Available Treatment Options

Gene	Protein	Disease	Year(s)	Reference No.	Inheritance	Main Treatment Options	Notes
GAA	Glucosidase alpha	Pompe disease	1986–1990	24, 25, 26	AR	Enzyme‐replacement therapy, noninvasive ventilation	a
GLA	Galactosidase alpha	Anderson‐Fabry disease	1989–1994	27, 28, 29	X	Antiplatelet/anticoagulant agents, enzyme‐replacement therapy, analgesic drugs to relieve neuropathic pain	b
LAMP2	Lysosome‐associated membrane protein 2	Danon disease	2004–2007	30, 31, 32	X	ICD implantation	c
PRKAG2	Protein kinase AMP‐activated Non‐catalytic subunit gamma 2	Wolff‐Parkinson‐White syndrome	2001	33	AD	Antiarrhythmic drugs, ablation	d
TTR	Transthyretin	Transthyrethin amyloidosis	1991–2002	34, 35, 36, 37	AD	Liver/kidney/heart transplantation	e

Of note, 15 other genes (including RASopathy genes such as PTPN11 and RAF1) have been classified as with ≥ moderate evidence by ClinGen for syndromic conditions where HCM can be seen. RASopathy genes are not included in the “genocopies of HCM” category as diagnostic discrimination between RASopathies and HCM is usually easier, due to the systemic features of the former (although in rare cases they may still resemble isolated HCM). AD indicates autosomal dominant; AR, autosomal recessive; HCM, hypertrophic cardiomyopathy; ICD, implantable cardioverter‐defibrillator; and X, X‐linked.

a Other reports^38^, ^39^, ^40^ determined the gene sequence and contributed to show that the disease was due to a lack of alpha‐glucosidase.

b Other reports^41^, ^42^ determined the gene sequence and contributed to show that the disease was due to a lack of alpha‐galactosidase.

c Reports by Danon et al^43^ and Nishino et al^44^ described Danon disease as a distinct lysosomal glycogen storage disease and showed that the cause was a deficiency of lysosome‐associated membrane protein 2.

d The reported study (Gollob et al^33^) consisted of a linkage analysis on two pedigrees including 70 individuals, with a LOD score for co‐segregation of variants in PRKAG2 and disease of 9.82.

e A large number of studies contributed to characterize transthyretin (eg, refs. 45, 46). The gene localization was determined by Jinno et al^47^.

**Figure 1:**
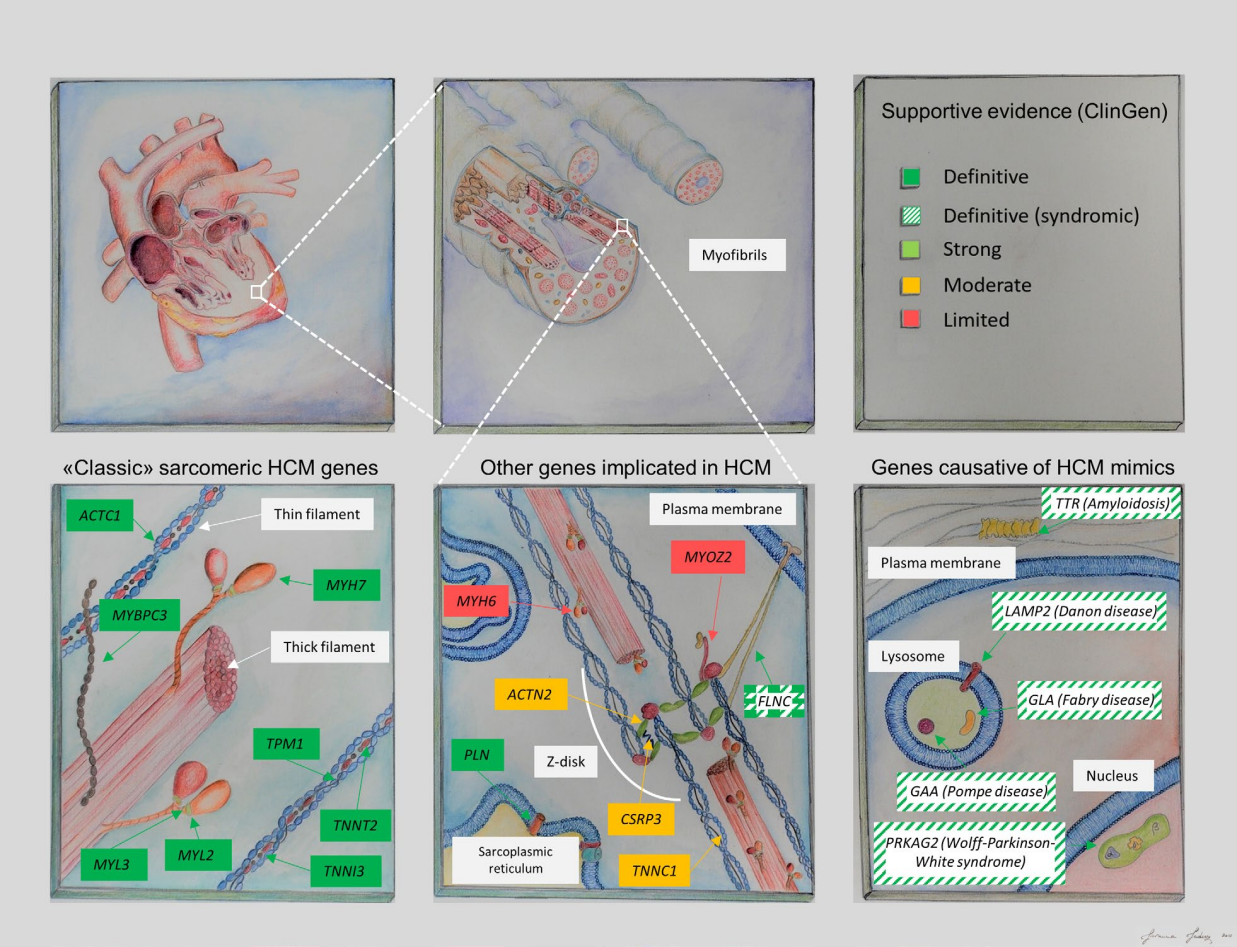
Examples of molecular complexes and proteins of the cardiomyocyte encoded by genes associated with sarcomeric HCM, its mimics and syndromic conditions featuring HCM with different levels of supporting evidence. Strength of evidence in favor of pathogenicity (top right panel) is coded as in the curation effort by the ClinGen consortium.^48^ Details about genetic associations for definitive genes displayed in the bottom left and bottom right panel, and genes with ≥ moderate supportive evidence are provided in Tables 1, 2 through 3. HCM, hypertrophic cardiomyopathy.

Improvements in sequencing technologies and their throughput, particularly with the advent of next‐generation sequencing (NGS), revolutionized the ability to investigate the genetic architecture of Mendelian diseases such as HCM. Case‐control rare variant comparisons became the design of choice for most studies, where usually relatively small groups of patients and controls were sequenced on one or a few genes of interest, encoding proteins within or outside the sarcomere, implicated in HCM with varying levels of supportive evidence^48^, ^49^ (Figure 1).

Whenever a protein‐altering variant was observed uniquely in patients, the absence of such variants in controls was often used as stand‐alone evidence in support of the claim for pathogenicity, with little convincing segregation or functional evidence in support. Although at the time these approaches were generally considered sufficient to implicate genes in Mendelian disease, it is now recognized that more substantial evidence is necessary to demonstrate association between variants in a given gene and disease.

**Figure 2:**
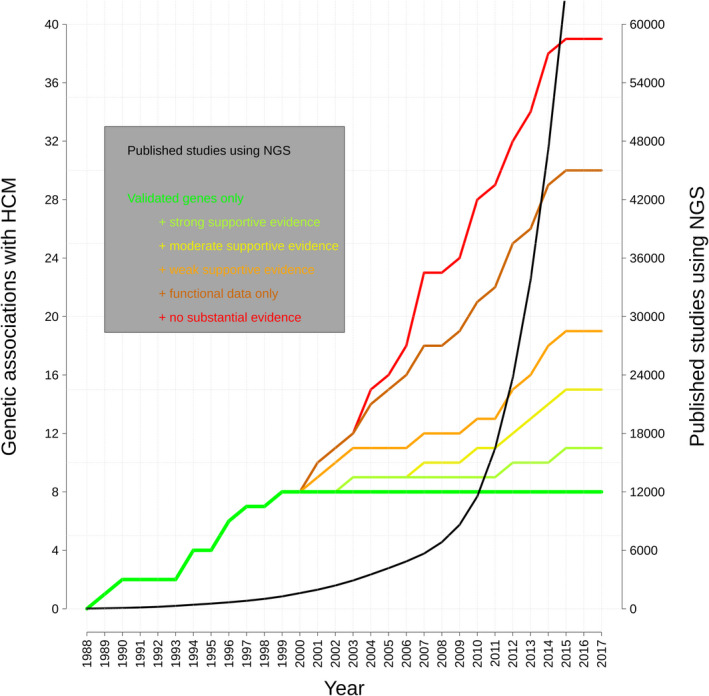
Number of genes implicated in HCM in the literature with different levels of supporting evidence (black line, left *y* axis) ranging from fully validated genes (darker green line) to those without substantial evidence supporting pathogenicity. The black line indicates the number of scientific publications in PubMed featuring next‐generation sequencing (colored lines, right *y* axis). The classification criteria for evidence in favor of pathogenicity (as detailed in the legend) and the genes included (n=39) reflect the analysis and curation effort by Walsh et al.^49^ HCM, hypertrophic cardiomyopathy; and NGS, next‐generation sequencing.

The consequence of these efforts was an exponential increase in the number of proposed genetic associations in the years between 2000 and 2015 (Figure 2). By 2016, 64 genes had been claimed to be causative in HCM (Human Gene Mutation Database v.2016.3, Table S1) with few of the additional genes being supported by sufficiently comprehensive and robust evidence^50^, ^51^, ^52^, ^53^, ^54^, ^55^, ^56^, ^57^ (Table 3).

**Table 3:** Other Genes Classified as With Moderate/Strong/Definitive Evidence for Isolated HCM (or Multiple Conditions Including Isolated HCM) by ClinGen,^48^ or With Convincing Evidence for HCM Causation Published After the ClinGen Curation Effort

Gene	Protein	Disease	Year	Reference No.	Inheritance	ClinGen Classification	Notes
TNNC1	Troponin C type 1 (slow)	Isolated HCM	2001	36	AD	Moderate	···
PLN	Phospholamban	HCM, DCM, and ARVC	2003	37	AD	Definitive	···
CSRP3	Cysteine and glycine‐rich protein 3 (cardiac LIM protein)	Isolated HCM	2003	38	AD	Moderate	···
JPH2	Junctophilin 2	Isolated HCM	2007	39	AD	Moderate	···
ACTN2	Actinin, alpha 2	HCM, LVH, LVNC, DCM, idiopathic VF	2010	40	AD	Moderate	···
FLNC	Filamin C, gamma	HCM, myofibrillar myopathy	2014	41	AD	Definitive	···
ALPK3	Alpha‐kinase 3	HCM, DCM (infant‐onset)	2016	42	AR	Strong	···
FHOD3	Formin homology 2 domain containing 3	HCM	2018	43	AD	···	a

The year and reference reported refer to the main publication in which a disease‐causing role in HCM was proposed. AD indicates autosomal dominant; ARVC, arrhythmogenic right‐ventricular cardiomyopathy; DCM, dilated cardiomyopathy; HCM, hypertrophic cardiomyopathy; LVH, left‐ventricular hypertrophy; LVNC, left‐ventricular non compaction; and VF, ventricular fibrillation.

a FHOD3 was not curated by ClinGen due to the later publication of the reported study (Ochoa et al^57^). Authors demonstrate a significant excess of rare variants in FHOD3 compared with controls and provide evidence for co‐segregation of FHOD3 variants with HCM using 17 pedigrees (combined LOD score=7.92).

Population databases of whole‐exome sequencing (WES)—and in part, whole‐genome sequencing (WGS)—such as the 1000 Genomes Project^58^ (≈2500 individuals) and National Heart, Lung, and Blood Institute Exome Sequencing Project^59^ (ESP, ≈6500 individuals) enabled early observations that many variants previously associated with cardiomyopathies were instead likely benign, as their population frequencies were incompatible with the prevalence of disease.^60^, ^61^ The definitive proof of this arrived in October 2014 with the release of the Exome Aggregation Consortium (ExAC) data set, a large collation of WES data from over 60 000 individuals,^62^ in which the average individual was shown to carry ≈54 variants previously reported as pathogenic in widely used databases of disease‐causing variants.

In the case of HCM and dilated cardiomyopathy (DCM), large‐scale analysis based on ExAC demonstrated that the frequency of rare variants found in many genes implicated in the condition was comparable to background population variation,^49^, ^63^, ^64^ casting doubts on the veracity of many purported associations (Figure 2). In addition, a number of independent disease‐ and gene‐specific approaches have recently been developed to aid decisions on which genes to include in clinical diagnostic panels. One recent example is the *diagnostic effectiveness* (*DEff*),^65^ a gene‐ and disease‐specific metric combining measures of actionability for cascade screening and prior likelihood of pathogenicity of variation detected in patients. *DEff* scores of validated and putative disease genes can be compared, in order to assist the decision on which of the latter should be routinely assessed in clinical practice and which would yield, at most, inconclusive results (variants of uncertain significance).

Rigorous, disease‐specific gene curation efforts that assess all available lines of evidence for disease association are now essential for genetically heterogeneous Mendelian diseases like HCM. In spite of the lack of a standardized approach to weight different types of evidence for gene‐disease associations, results obtained by curation/classification efforts are characterized by general concordance, with the 8 validated genes unanimously considered as incontrovertibly associated with HCM, and genes such as *PLN* and *CSRP3* supported by strong evidence in favor of HCM pathogenicity.^48^, ^49^, ^65^ The ClinGen initiative^66^ (https://clinicalgenome.org/)—founded to define the clinical relevance of genes and genetic variants—has recently established a framework to systematically assess all gene‐disease claimed associations’ veracity and their clinical validity based on published evidence. The curation effort for HCM, assessing >50 genes, has recently classified evidence in support of their role in HCM as “definitive” for the 8 validated sarcomeric genes, *PLN* and *FLNC* (also associated with myofibrillar myopathy), “strong” for *ALPK3* and “moderate” for *ACTN2*,* CSRP3*,* TNNC1* and *JPH2* (Tables 1 and 3).^48^ Other 20 genes were assigned a ≥ moderate evidence classification for their role in genocopies of HCM (Table 2) or syndromes involving HCM.

## Genetic Testing Strategies

The primary purpose of genetic testing in HCM is to facilitate cascade screening in relatives of the index patient in order to identify those at risk and those not at risk of developing disease. As guidelines currently recommend regular clinical evaluation for first‐degree relatives of all HCM patients,^2^ discharging those relatives lacking identified pathogenic variants offers obvious psychological benefits for these individuals and significant cost savings for healthcare providers. Genetic testing for HCM has been shown to be more cost effective than clinical screening alone in UK^67^ and Australian^68^ studies, and the Partners Laboratory for Molecular Medicine (LMM) estimated savings of USD 700 000 (based on Medicare rates) from discharging genotype‐negative relatives of 2912 HCM probands.^69^ Ongoing reductions in the costs of DNA sequencing, and improvements in variant analysis as described subsequently, will further support the economic case for HCM genetic testing. It has also been recognized that genetic testing in probands can increase the often low take‐up in clinical screening from at‐risk relatives.^70^

In contrast, knowledge of the causative genetic variant does not currently affect the clinical management for most HCM patients and is not included in sudden cardiac death (SCD) risk assessment models.^71^, ^72^ The exception to this is identifying patients with pathogenic variants in a small group of genes—such as *GAA*,* GLA*,* LAMP2*,* PRKAG2* and *TTR*—that are incontrovertibly associated with metabolic diseases that mimic HCM (Figure 1 and Table 2) but have distinctive clinical profiles, inheritance patterns, and treatment options.^73^ The inclusion of these genes in diagnostic panels for HCM is advantageous given their pronounced phenotypic similarity with the classic sarcomeric form^74^ and the importance of a prompt differential diagnosis, enabling correct treatment decisions and optimal patient management and de facto representing a significant step toward more personalized approaches.

Despite limited evidence for a causal role in HCM, many of the non‐sarcomeric genes implicated in the disease have been progressively included in panels developed by clinical laboratories and commercial providers^75^ (listed in the National Center for Biotechnology Information Genetic Testing Registry: https://www.ncbi.nlm.nih.gov/gtr/). The uncertainty around the pathogenicity of these “genes of uncertain significance” in HCM has led to difficulty for clinical diagnostic laboratories in choosing which of the ambiguous genes to include in routinely used diagnostic panels. The choice ultimately lies on the spectrum between the extremes of including only the validated genes at one end (bottom left and bottom right panels in Figure 1) and systematically screening all genes implicated in HCM at the other, irrespective of the strength of evidence in support of their causal role for HCM. The former, conservative choice plausibly leads to the possibility of false negative genetic tests but the latter, inclusive option, bears greater potential for false positive results, which can be detrimental for the patients and their family members, and increases the uncertainty associated with testing through an epidemic of variants of unknown significance (VUS). Unsurprisingly, expanding HCM panels to include genes of questionable pathogenicity has had little effect on the sensitivity of genetic testing. For example, LMM reported identifying only one pathogenic variant in 632 cases (in the *PLN* gene) outside of the 8 core sarcomeric genes and metabolic cardiomyopathy genes.^69^

Ongoing reductions in the cost of sequencing have meant that WGS (complete sequencing of the entire genome in an individual) and WES (sequencing of the 1% of the genome that encodes proteins) may become feasible options as first‐line sequencing assays in the near future. However, there is limited evidence that WGS and WES are currently cost effective in clinical practice.^76^ For example, a recent study that was part of the Medseq initiative to evaluate the clinical potential of WGS^77^ compared the performance of a targeted gene panel and WGS in 41 HCM patients. WGS identified 19 of the 20 variants of interest detected by panel sequencing, but missed a 18 bp duplication in *MYBPC3* due to low coverage and only additionally identified a causative variant in a RASopathy gene (not in the panel) as well as several VUS and secondary findings. The limited value of WGS, WES, or super‐sized gene panels for standard HCM genetic testing, that is, adult‐onset and/or autosomal dominant disease in the absence of large family pedigrees, is clear when considering current variant interpretation guidelines for clinical testing and the requirements for defining a variant as actionable (see next section). It will be impossible to classify a variant in a novel gene as clinically actionable in the vast majority of cases, regardless of how plausible that candidate gene may be, as the guidelines require as a minimum that the gene or variant class has a proven role in the disease.

In contrast, WGS/WES can offer some advantages as diagnostic sequencing assays if testing is restricted to genes and regions that have been validated for the disease in question. In this “virtual panel testing” approach, analysis is restricted to only those genes that can yield interpretable variants, but the extensive sequencing enables reanalysis of archived data as new and validated gene‐disease associations are published, as well as potentially generating comprehensive and useful data sets for research purposes. One advantage of WGS compared with approaches targeting only protein‐coding regions of genes (eg, targeted gene panel and WES) is its enhanced ability to identify variants classes such as copy number variants (CNVs)—deletions or duplications of whole exons, genes, or chromosomal regions—and variants in noncoding regions of the genome such as introns. Studies exploring the role of CNVs in HCM have found such variation in ≈1% of cases,^78^, ^79^, ^80^ although detecting, validating, and interpreting CNVs remains a challenging and nonstandardized task. A recent study used WGS to identify deep intronic variants in *MYBPC3* in 4 HCM patients who were negative for standard genetic testing, and these variants were shown to affect splicing and produce truncated transcripts.^81^ The contribution of these noncoding variants that affect gene dosage is likely to be particularly relevant to genes where the underlying disease mechanism is haploinsufficiency (ie, where the loss of half of the physiological amount of functional protein causes disease). This is often the case for genes in which protein‐truncating variants are the most prevalent pathogenic variant class although, in the case of *MYBPC3*, haploinsufficiency has also been proposed as the molecular mechanism for several missense variants.^82^ For most of the other major sarcomeric HCM genes where variants act in a dominant negative manner, such noncoding variants are expected to be less relevant.

In addition to these scientific pros and cons, other factors such as affordability and the quality and technical features of generated data are also important factors in choosing a sequencing platform for genetic testing.^83^, ^84^ As highlighted previously, depth of coverage for affordable WGS/WES in the diagnostic setting may currently be inferior to panel sequencing and lead to false negative findings—this is particularly relevant for WES assays as some exons, especially in GC‐rich regions, are not adequately captured.^85^ It is also important to recognize the extra burden of data processing, analysis, and storage associated with WGS/WES, as well as ethical issues relating to secondary and incidental findings if analysis is not restricted to valid disease genes for the primary condition.

**Figure 3:**
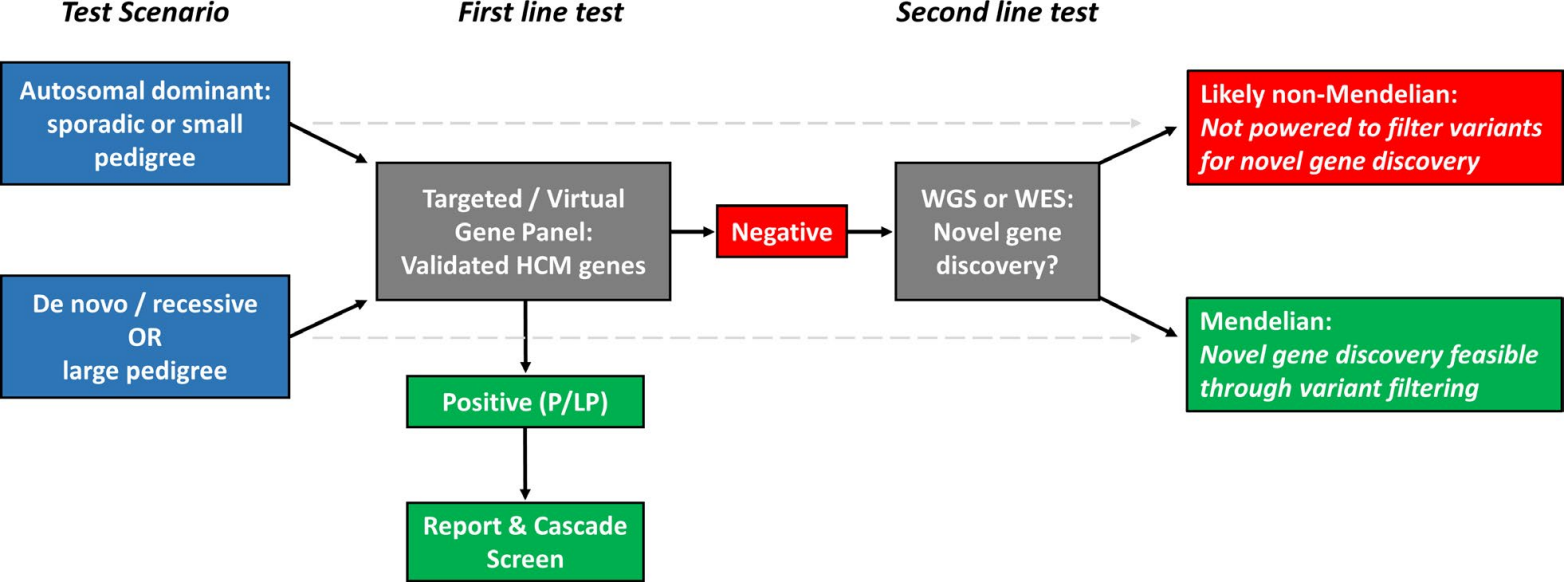
Proposal for a tiered approach to genetic testing in HCM. Initial testing is restricted to validated HCM genes using either a targeted gene panel or a virtual panel by focused analysis of WGS or WES data. The latter allows for reanalysis of genes subsequently linked to HCM, and WGS can detect additional variant classes including CNVs and deep intronic splice variants, though sequencing coverage is likely to be inferior to targeted panel sequencing. In the case of negative results, variant prioritization in the research setting can aid identification of the causative variant, directly translating into a diagnostic finding. The likelihood of detecting variation in novel genes with broader WGS/WES analysis depends on the profile of the patient and family being tested. In general, large pedigrees informative for segregation and trios (affected child with unaffected parents, indicating likely de novo variant occurrence or recessive inheritance) can enable filtering to a manageably small number of potentially causative variants for detailed evaluation. CNV, copy number variant; HCM, hypertrophic cardiomyopathy; WES, whole‐exome sequencing; and WGS, whole‐genome sequencing.

At present, the primary role for unbiased WGS/WES lies in the research setting in order to identify putative causative variants outside of known HCM genes in cases with an inheritance pattern that enables triage and prioritization of rare variants and where the genetic cause of disease remains elusive after initial genetic testing. These cases include autosomal dominant HCM in large and clinically defined family pedigrees, although such cases are rarely encountered during routine clinical genetic testing. More commonly, novel genes underlying recessive or de novo inheritance in pediatric cases can be identified through sequencing of the patient and their unaffected parents (trio analysis) as well as other family members if available. If a definitive genetic cause is identified, such a research finding is of direct diagnostic utility. These approaches have recently identified recessive truncating variants in the *ALPK3* gene as pathogenic in several pediatric cardiomyopathy cases.^56^ Taking all of these issues into consideration, an effective tiered approach to genetic testing in HCM is shown in Figure 3.

Analysis should be initially focused on validated HCM genes, regardless of the sequencing assay applied (gene panel or focused analysis of WGS/WES). If negative, the decision to expand analysis to novel genes (with WGS or WES) would depend on the profile of the patient being tested and the likelihood of isolating the causative variant from genome‐wide sequencing. For most adult probands, it is unlikely that pathogenic variants will be identified in genes not previously associated with HCM. In any case, as described subsequently, it is increasingly likely that most such patients represent non‐Mendelian and nonfamilial HCM.

## Interpretation of Genetic Findings

**Figure 4:**
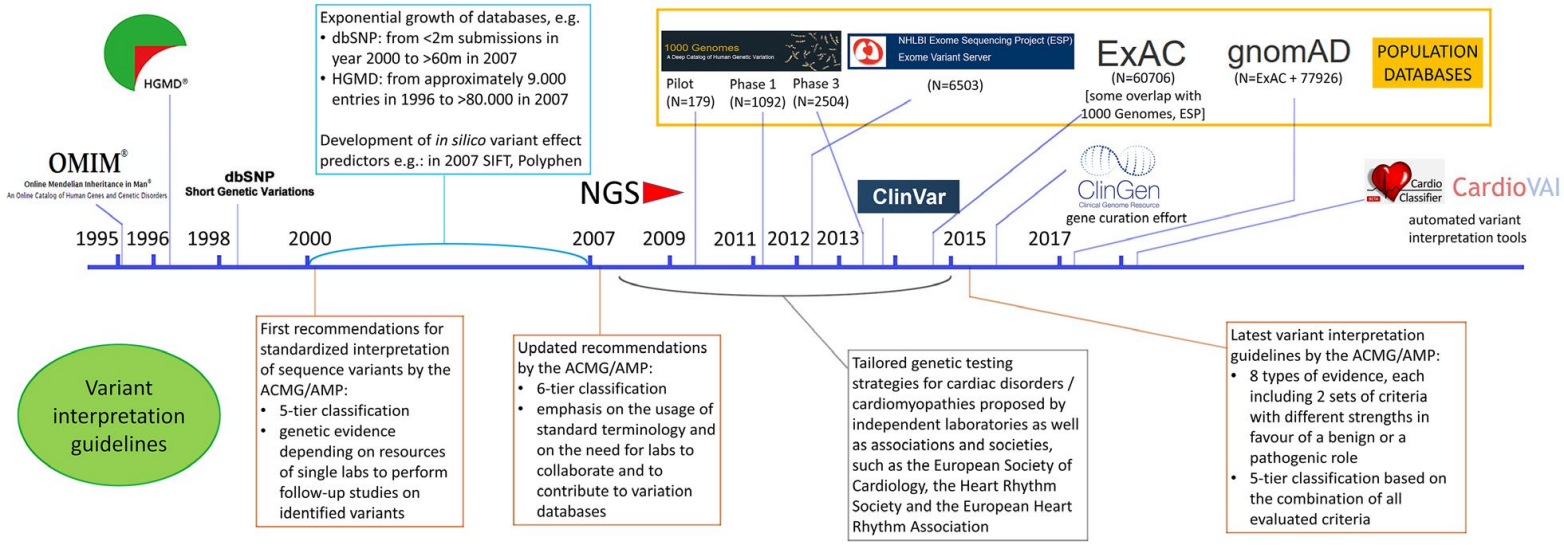
Variant interpretation guidelines issued by the ACMG/AMP and independent laboratories/societies/associations (below the timeline) and some of the main resources contributing to a finer interpretation of sequence variants (above the timeline) as they became available. Several sequence variation databases with a focus on variants’ role in disease were released in time, particularly in the late 1990s (eg, the Human Gene Mutation Database in 1996) but also in more recent years (eg, ClinVar in 2013). The advent of cost‐effective, high‐throughput NGS in the years between 2005 and 2010 enabled the establishment of progressively large genome‐wide population variation databases, starting with the Pilot data set of the 1000 Genomes Project in 2009. All these increasingly complex and large‐scale resources on one hand allow a much more detailed characterization of genetic variants, but on the other contribute to the constant growth in the variability and quantity of data types and information on single variants, variant classes, and genes and require increasingly articulated guidelines for variant interpretation. Several efforts are in place to finely tune variant interpretation in a gene‐ and disease‐specific manner (eg, gene curation efforts such as ClinGen,^80^ established in 2015) and to render interpretation easier and quicker for the geneticist with automated variant interpretation tools (eg, CardioClassifier^92^ and CardioVAI,^93^ that interpret variants in the context of cardiomyopathies). ACMG, American College of Medical Genetics; AMP, Association for Molecular Pathology; and NGS, next‐generation sequencing.

Determination of the clinical significance of genetic variants is a complex process involving the evaluation and weighting of several lines of evidence. Accurate interpretation is key, given the potentially detrimental consequences that an erroneous interpretation can have on the proband and their relatives.^86^, ^87^ The American College of Medical Genetics and the Association of Molecular Pathologists (ACMG/AMP) first issued standardized recommendations on the interpretation of sequence variation almost 2 decades ago,^88^ and a series of updated and increasingly articulated guidelines have been issued in the following years, in parallel with the establishment and the exponential growth of clinical and population sequence variation databases (Figure 4).

The increasing complexity of variant interpretation and the extent of misclassification in previous years underscored the need for more stringent and comprehensive approaches to variant classification, prompting the release of the current ACMG/AMP guidelines in 2015^89^ (Figure 4). These guidelines, considerably more complex than previous recommendations, are designed to be applicable to variants in all Mendelian genes, and to serve as a basis for more detailed and focused implementation for specific genes and diseases. As a result, several rules embedded in the current interpretation framework avoid providing specific instructions on how to weight certain classes of evidence, inevitably giving rise to differences in approaches adopted by different laboratories and discordant variant classification.^90^, ^91^ For this reason, besides the development of automated, guideline‐based variant interpretation tools,^92^, ^93^ the implementation of consensus guideline adaptations is now an active area of research, both for different evidence categories and for specific disorders or gene‐disease pairs. Examples include proposals for weighting the evidence of co‐segregation of variants with disease in pedigrees^94^ and for a more finely tuned interpretation of loss‐of‐function variants,^95^ quantitative scores estimating the likelihood of pathogenicity based on the variant location within the protein sequence,^96^ an algorithm to define a disease‐specific population frequency cut‐off for potentially pathogenic variants.^97^ More complex approaches taking into account the variant position within the three‐dimensional structure of the protein (as opposed to its one‐dimensional sequence) have also been used to identify protein regions enriched in HCM‐causing variants,^98^ and could augment variant interpretation accuracy if embedded in the variant interpretation framework. For specific genes/diseases, tailored recommendations to interpret genetic variation have been developed for RASopathies^99^ and for *MYH7* variants in HCM,^100^ with ongoing efforts for other genes and diseases by the ClinGen initiative and other groups. A description of ACMG/AMP guidelines’ rule‐specific application to variants detected in HCM patients, current issues, and ongoing/future developments toward an improved application are provided in Table 4.

**Table 4:** Summary of the Main Lines of Evidence Used to Assess the Pathogenicity of Genetic Variants as Described by the ACMG/AMP Variant Interpretation Guidelines, Including Their Specific Application to Variants Detected in HCM Patients, Alongside Current Criteria‐Specific Issues and Ongoing/Future Developments for a More Refined Application

ACMG/AMP Rule/Evidence Class	Application to HCM	Issues and Future Developments
PVS1—null/truncating variant in gene with loss‐of‐function mechanism for disease.	Applied to truncating variants in *MYBPC3*, detected in ≈10% of HCM patients.	Some variants may not lead to nonsense‐mediated decay and haploinsufficiency.^95^ Noncoding variants that may lead to a truncated transcript (eg, splicing variants) can be difficult to detect and interpret.
PS1/PM5—same amino acid change/change at same residue as an established pathogenic variant	Numerous established pathogenic HCM variants and many examples of different variants affecting same residues.	Difficult to unambiguously define what is an established pathogenic variant. Curation of ClinVar entries is ongoing and will create a high confidence set of the most common pathogenic variants.
PS3—proven deleterious effect with functional studies.	Animal or cell‐based studies can be used to assess the phenotypic effect of a variant detected in a patient.	Uncertainty about the translatability of evidence from in vitro or in vivo models to the clinical setting. Currently impractical for regular application in a clinical genetic setting. High throughput assays with demonstrated translatability for known pathogenic and benign variants could produce a valuable database for all possible single nucleotide variants in sarcomeric genes.
PS4—variant is significantly enriched in cases compared with controls.	There are numerous founder and recurrent pathogenic HCM variants that are observed in multiple HCM probands/families. Comparison with control or population data sets can identify significantly enriched variants.	Guidelines for *MYH7* variants suggest presence in distinct numbers of HCM probands for strong (≥15), moderate (≥6) or supporting (≥2) evidence.^100^ However, this does not demonstrate statistically significant enrichment in cases, for which large case and control/population data sets are required. The threshold for defining a significant enrichment and methods for dealing with multiple testing in cohort studies need to be addressed. Most currently available data sets are derived from European ancestry populations that are unlikely to include recurrent variants from other population groups.
PM1/PP2—relative frequency of variants in cases and controls for genes or gene regions.	All sarcomeric genes enriched for rare variants in HCM, with several mutation hotspots, eg, *MYH7* head domain.	Current rules are ambiguously defined and not based on quantitative measures. Methods now developed based on case‐control analysis and definition of enriched clusters provide a quantitative approach, with evidence strength dependent on level of enrichment in cases.^96^
PM2—variant is rare enough in the population to be plausibly pathogenic (also BA1, BS1, BS2).	Population frequency data from gnomAD and disease‐specific threshold based on HCM characteristics provide stringent variant rarity threshold.^97^	Low penetrance variants or modifiers may be less rare in the population than standard pathogenic variants, requiring evidence from other rules to achieve (likely) pathogenic classification. Some population groups are still not well represented in databases like gnomAD (eg, North Africa and West Asia), variants from patients from these groups need to be analyzed with caution.
PP1—segregation of variant with disease in family pedigrees.	Segregation evidence is available for many HCM‐causing variants (in literature and ClinVar). Strength applied to evidence depends on the number of informative meioses.^94^, ^100^	As many HCM variants are private or detected in small family pedigrees, this evidence class will not be informative for a large proportion of variants. Incomplete penetrance in HCM means phenotype‐negative variant carriers are uninformative for pedigree analysis. Data for this evidence can be difficult to derive from literature and ClinVar.
PP3—computational evidence to support a deleterious effect.	As with most other Mendelian diseases, predictions from several different algorithms are used to provide supportive evidence for pathogenicity.	Algorithms lack specificity and therefore provide limited supportive evidence. Consensus findings from several orthogonal techniques should be used but there are numerous available algorithms and little agreement on the most appropriate subset of algorithms to use. Algorithms are generically applied to all genes but may not be equally effective.

ACMG indicates American College of Medical Genetics; AMP, Association of Molecular Pathologists; and HCM, indicates hypertrophic cardiomyopathy.

The current guidelines are designed to minimize false‐positive results (ie, over‐calling of variant pathogenicity) as it is usually less detrimental for patients and their relatives to receive an inconclusive result in presence of a pathogenic variant than it is to receive a positive result in absence of a real genetic cause. While assigning variants a categorical classification, guidelines essentially follow an underlying Bayesian framework, with a variant considered “likely pathogenic” if its probability of pathogenicity is ≥90%, and “pathogenic” if ≥99%.^101^ However, the lack of variant‐specific information available to diagnostic laboratories for many rare novel variants often translates into the inability to correctly identify those with a probability of pathogenicity ≥90%, de facto rendering the application of the guidelines very stringent and causing a general under‐calling of pathogenicity.

Although this conservative approach offers the advantage of minimizing false‐positive variant classifications, this inevitably comes at the cost of an increased rate of false negative results (ie, where pathogenic variants are classified as VUS). This drawback is particularly relevant in HCM because of its marked genetic heterogeneity with many variants private to affected families, the fact that most causative variants are missense substitutions which are inherently more difficult to interpret than protein‐truncating variants and occur with higher background rates in controls, and the limitations of functional assays to effectively evaluate their molecular effects unlike, for example, those in genes encoding ion channels. Recent evidence has quantified this false negative rate for HCM, suggesting that ≈8% of HCM patients who undergo clinical genetic testing will receive a reported VUS that is likely to be a pathogenic variant^63^ (ie, a VUS in one of the 8 sarcomeric genes was detected in 12.4% of HCM patients compared with a background rate of rare variation of 4.3% in the ExAC population data set). In support of this, analysis of the SHaRe registry (https://theshareregistry.org/), comprising genetic data and cardiac morphofunctional parameters for >9000 HCM patients), showed that variants classified as VUS have a measurable clinical impact on outcome,^102^ highlighting how these include misclassified pathogenic variants.

In order to overcome these obstacles to variant interpretation, knowledge sharing between different centers through databases such as ClinVar (https://www.ncbi.nlm.nih.gov/clinvar/) is critical. As an example, the *MYH7* p.Arg869His variant had conflicting interpretations in ClinVar (VUS or likely pathogenic) based on the variant characteristics and some published reports. However, this variant is observed at locally high frequencies in northern Tuscany (detected in 30 of 1198 unrelated HCM probands tested at Careggi University Hospital compared with 0 of 356 non‐HCM patients affected with other cardiovascular conditions, *P*=6.22×10^−4^), including co‐segregation with disease in 7 HCM patients in a three‐generation family. Based on this evidence (detailed in ClinVar accession SCV001147002.1), the *MYH7* p.Arg869His variant can now be classified as definitively pathogenic for HCM and highlights the benefits of sharing findings from different populations and geographic regions.

The role of both public and private diagnostic laboratories is key, as they represent the “hubs” where many novel variants are observed and where patients and their families are referred when a genetic cause of overt or suspected disease is investigated. Many diagnostic laboratories (such as Invitae and GeneDX) comprehensively publish variant interpretation summaries in ClinVar, based on published data and in‐house findings, which helped ClinVar recently reach the milestone of 1 million submissions. Nevertheless, some diagnostic companies still refuse to share data in this manner and instead produce proprietary variant interpretation that takes advantage of public resources such as ClinVar and gnomAD in addition to their own databases. Given the arduous challenge of variant interpretation for genetically heterogeneous diseases like HCM, and the importance of accurate classification in clinical practice, the ethics of such practices are dubious. One possible solution would be for healthcare providers and cardiologists/clinical geneticists to insist on submission to ClinVar when engaging the services of diagnostic laboratories or only employ companies committed to knowledge sharing.

Data sharing initiatives are at the basis of several approaches that aim to tackle the high rate of inconclusive findings in clinical genetic testing. The availability of increasingly large patient and control cohorts may enable the development of quantitative statistical approaches to identify variant classes with high likelihood of pathogenicity. For example, case‐control analyses can estimate these prior probabilities of pathogenicity through metrics such as the odds ratio and *etiological fraction*.^63^ A recent study using this approach, which defined regions of genes where case variants are significantly enriched and incorporated this evidence to the ACMG/AMP framework, increased the relative yield of clinically actionable variants in sarcomeric genes by up to 20% in HCM.^96^ More comprehensive classification algorithms based on ensemble‐ or machine‐learning techniques that integrate different classes of evidence may also plausibly improve the accuracy of variant classification and contribute to lowering the proportion of inconclusive findings in HCM and other diseases.^103^, ^104^ Advanced computational techniques like these may face considerable hurdles though with integration into current guidelines as well as the workflows of clinical genetics laboratories.

Assays that accurately assess the functional effects of specific genetic variants, through cell‐based or in vivo models, also offer the possibility of informing the pathogenicity of variants without the need for segregation evidence from large families. However, the lack of effective and standardized translational assays has limited the role for this class of evidence for cardiomyopathies, with the cost and time to implement such assays also rendering them impractical in the context of clinical genetic screening. Novel techniques, in particular those using the CRISPR/Cas9 genome editing technology, are now being applied for distinct alleles in HCM^105^, ^106^, ^107^, ^108^ and DCM.^109^ The further development of such approaches into more comprehensive and high‐throughput assays, such as a recent study that functionally evaluated thousands of alleles in *BRCA1* 110 simultaneously, may be required for such evidence to be routinely incorporated into clinical genetic testing.

Improvements in variant classification are especially relevant to non‐white populations, which are characterized by significantly lower detection rates of (likely) pathogenic variants in cardiomyopathy patients compared with white patients.^111^ This is likely to result from the fact that the vast majority of research efforts and genetic tests for cardiomyopathies have been carried out on patients and controls of European descent (and therefore recurrent and founder variants occurring in whites are more likely to have been previously observed and characterized). This is emblematic of a wider problem in genetics research, exemplified by the proportion of genome‐wide association studies (GWAS) performed on individuals of European descent (≈85% of the total) being vastly disproportionate to their proportion of the world population (≈15%).^112^ These observations underscore the urgent need for more genetic research to be undertaken in non‐European populations as well as the further development of functional and quantitative approaches that reduce reliance on the prior observation and characterization of rare pathogenic variants.

The accurate interpretation of variants in HCM genes is equally critical when they have been detected as secondary or incidental findings by WGS or WES in individuals undergoing genetic testing for noncardiac conditions. Each of the 8 core sarcomeric HCM genes are on the list of genes recommended by the ACMG to be analyzed as secondary findings.^113^ Given the relatively high rate of rare variation in these genes in the population, only those variants with strong evidence of pathogenicity should be considered actionable. However, as many HCM variants display reduced penetrance, clinical evaluation to detect the cardiomyopathy phenotype is essential, including ongoing surveillance screening where appropriate.^114^

## Interpretation of Genotype‐Negative HCM

When variants in sarcomeric genes were first implicated in HCM, there were indications that knowledge of the causative variant might be prognostic for patients and potentially useful for clinical decision‐making. For example, *TNNT2* variants were initially shown to be associated with poor prognosis and high incidence of sudden cardiac death (SCD),^115^ whereas variants in *MYBPC3* were related with delayed onset of disease and more favorable outcomes.^116^ Subsequently, genotype‐phenotype analyses on ever‐larger cohorts of HCM patients have been undertaken to clarify these initial findings and attempt to identify clear and consistent correlations between the pathogenic variant class and the clinical profile and outcomes in patients.

Recent cohort studies have identified some statistically significant differences in phenotype between patients carrying different variant classes. In particular, HCM patients with variants in thin filament genes (*TNNT2*,* TNNI3*,* TPM1*,* ACTC1*) were shown to have distinctive patterns of left‐ventricular hypertrophy and diastolic profile, as well as lower prevalence of left‐ventricular outflow tract obstruction (LVOTO) and late gadolinium enhancement (a marker of myocardial fibrosis) compared with those with variants in thick filament genes (*MYH7*,* MYBPC3*).^117^ Although patients with variants in thin filament genes were more likely to develop advanced LV dysfunction, no differences were observed in mortality or SCD between thin and thick filament variant‐positive patient groups in this study.^117^

Studies comparing outcomes between patients with *MYH7* and *MYBPC3* pathogenic variants (the predominant genes associated with HCM) have yielded conflicting results—no significant differences were observed for 2 moderately sized cohorts^118^, ^119^ but in the larger SHaRe registry those with *MYH7* variants had a higher risk of advanced heart failure and overall composite outcome.^102^ Furthermore, patients with complex genotypes carrying 2 pathogenic mutations were at greater risk of adverse outcome and sudden cardiac death, irrespective of the genes involved. Despite these broad correlations, all of these studies have revealed extensive heterogeneity in symptoms, disease severity, and outcome, even among patients with the same or similar pathogenic variants. As a result, the individual patient's genetic status has limited impact on clinical management and is largely limited to cascade screening and to the small subsets of patients with rare storage or metabolic diseases mimicking HCM.

In contrast, clear and consistent differences in demographics, phenotype, and outcomes have been observed when comparing genotype‐positive and genotype‐negative patients, that is, those with and without causative variants in sarcomeric genes^102^, ^118^, ^120^, ^121^, ^122^, ^123^, ^124^, ^125^, ^126^ (Table 5).

**Table 5:** Summary of Demographic, Baseline Clinical and Outcome Data for Major Published HCM Studies Comparing Genotype‐Positive and Genotype‐Negative Patients

Center	SHaRe Registry	Mayo Clinic, USA	UCL, UK	Toronto General Hospital, Canada	Erasmus Medical Centre, Netherlands	Centenary Institute, Australia	Meta‐Analysis (13 Cohorts)
Study	Ho et al^102^	Bos et al^120^	Lopes et al^121^	Li et al^118^	van Velzen et al^125^	Ingles et al^126^	Lopes et al^124^
Cohort size	4591	1053	874	558	512	265	2459
Genes tested	8	9	8	8	Large panel	10	···
Genotype positive, %	46.3%	34.1%	43.8%	35.5%	45.7%	52.1%	···
Demographics							
Age at inclusion			45.8±14.7 vs 53.1±14.9		46±15 vs 55±15	47±16 vs 56±17	
			*P*<0.001		*P*<0.001	*P*<0.001	
Age at diagnosis	37.3±17.1 vs 49.0±17.4	36.4±17 vs 48.5±18		39.5±15.2 vs 48.5±14.8		34±17 vs 44±18	38.4±10.3 vs 46.0±10.4
	*P*<0.001	*P*<0.001		*P*<0.001		*P*<0.001	*P*<0.001
Sex (% male)	60.6% vs 66.0%	58.5% vs 60.4%		56.1% vs 70.3%	67.9% vs 61.5%	53.6% vs 70.9%	57.5% vs 61.5%
	*P*<0.01	*P*=0.6		*P*=0.001	*P*=0.13	*P*=0.005	*P*=0.422
FH HCM (%)	57.9% vs 24.5%	50.4% vs 22.9%	39.8% vs 15.6%	52.5% vs 20.0%		73.9% vs 30.7%	50.6% vs 23.1%
	*P*<0.001	*P*<0.001	*P*<0.001	*P*<0.001		*P*<0.001	*P*<0.001
FH SCD (%)		27.0% vs 15.0%	28.5% vs 15.2%	16.7% vs 7.2%	19.7% vs 5.4%	41.3% vs 6.3%	27.0% vs 14.9%
		*P*<0.001	*P*<0.001	*P*=0.002	*P*<0.001	*P*<0.001	*P*<0.001
Baseline characteristics							
Max LVWT, mm	19.7±6.2 vs 18.1±5.2	22.6±6 vs 20.1±5	18.8±4.4 vs 18.1±4.1	20.8±4.8 vs 19.6±4.9	20±5 vs 18±4	22±6 vs 21±5	21.0±4.1 vs 19.3±3.5
	*P*<0.001	*P*<0.001	*P*=0.004	*P*=0.16 (adjusted)	*P*<0.001	*P*=0.03	*P*=0.03
Hypertension (%)		19.2% vs 43.2%		25.3% vs 46.9%			22.8% vs 41.7%
		*P*<0.001		*P*<0.001			*P*=0.004
Clinical outcomes—hazard ratio (CI/log rank *P* value)							
Mean follow‐up years	5.4±6.9			6.6±6.3/6.2±5.6	12±9		
CV mortality	2.41 (1.73–3.35) (all death)		3.99 (*P*=0.001)		2.82 (*P*=0.002)		
HF‐related mortality					6.33 (*P*=0.004)		
SCD/aborted SCD			3.44 (*P*=0.028)		2.88 (*P*=0.015)		
Combined HF events	1.87 (1.55–2.25)			4.51 (*P*<0.001)			
Overall composite	1.98 (1.72–2.28)						

Genotype‐positive status is defined by the presence of a putatively pathogenic variant—the vast majority of these occur in one of the 8 core sarcomeric genes (*MYH7*,* MYBPC3*,* TNNT2*,* TNNI3*,* TPM1*,* MYL2*,* MYL3*,* ACTC1*). The meta‐analysis by Lopes et al^124^ involved 13 previously published cohorts—this included subsets of the cohorts from the Mayo Clinic and Toronto General Hospital that had been published prior to the larger versions of those cohorts described here. The SHaRe registry may include cases from the Erasmus Medical Centre study and the meta‐analysis by Lopes et al^124^. CI indicates confidence interval; FH, family history; HCM, hypertrophic cardiomyopathy; HF, heart failure; LVWT, left‐ventricular wall thickness; SCD, sudden cardiac death; and UCL, University College London.

Genotype‐positive patients present approximately a decade earlier and unsurprisingly have a greater proportion of family history of both HCM and SCD. Maximum left‐ventricular wall thickness (LVWT) is significantly greater in genotype‐positive patients, who are characterized by more asymmetrical hypertrophy than genotype‐negative cases. Most important, these differences translate into poorer outcomes for genotype‐positive patients across a range of measures and composites in a number of studies, including the 4591 cases of the SHaRe registry.^102^

In these studies, genotype‐negative cases were defined as lacking pathogenic (or potentially pathogenic, ie, rare and protein‐altering) variants in the tested HCM genes (for the most part the 8 validated sarcomeric genes). The expectation that at least some of these cases may have novel genetic causes drove the many attempts to discover novel HCM genes, as described previously. However, the non‐sarcomeric genes whose role in HCM has been validated thus far account for only a small proportion of HCM patients, with cumulative weight of evidence now suggesting that genotype‐negative HCM is often nonfamilial and most likely to represent non‐Mendelian disease.

Ingles et al^123^ recently defined “non‐familial HCM” as the condition that lacks both a potentially pathogenic sarcomeric variant (whether classified as pathogenic or VUS) and any prior family history for HCM. Nonfamilial cases (which accounted for 40% of the cohort in the study) were phenotypically distinct from genotype‐positive cases (though in a similar manner to overall genotype‐negative patients) and with very low yield from cardiac clinical screening on first‐degree relatives. Consistently, Ko et al^127^ found that only 3% of first‐degree relatives of “nonfamilial” probands were diagnosed with HCM by clinical screening, compared with 17% of relatives of probands with a positive genetic test and/or prior family history of HCM. As familial occurrence of HCM is still nonnegligible in pedigrees without an identified causative variant, current clinical recommendations state that regular clinical screening of family members should be considered for relatives in such families. However, the actual risk of disease in families without prior family history of HCM, and the consequent necessity for ongoing clinical screening in phenotype‐negative relatives, remains uncertain. In this respect, it is worth emphasizing that history taking in HCM is a complex task due to the high variability in terms of age of onset and phenotype severity characterizing this condition. In contrast to other conditions in which the phenotype usually manifests clearly and penetrance is not age related, in the case of HCM the adoption of specific strategies to maximize precision and completeness of the information gathered from the proband and his/her relatives may help to reconstruct a reliable clinical history of the family. As an example, in addition to applying the principles detailed in internationally accepted guidelines,^114^ at first clinical appointment the cardiologist could also ask for information regarding family history, so to complement and confirm those gathered by the genetic counsellor. Furthermore, it may be good practice to ask patients to share a copy of all clinical documents regarding important clinical events such as hospitalizations or cardiovascular events, due to the fact that the knowledge of family members on the cause underlying such events is generally limited.

Further large‐scale studies are required to definitively assess the risk in relatives of index cases with apparently nonfamilial HCM and to accurately define the criteria to establish family history of the disease. Such studies may lead to modification of current guidelines on the extent and frequency of clinical screening recommended for relatives of HCM patients, based on more accurate assessments of their risk of developing disease depending on genetic status and family history. This may offer the opportunity for additional significant savings in healthcare provision and will further underscore the utility and cost‐effectiveness of genetic testing for HCM.

## Future Directions

Almost 30 years after the first definitive evidence of the genetic etiology of HCM,^7^ great progress has been achieved and the genes underlying the majority of Mendelian monogenic cases of HCM have now been identified. It is clear that HCM remains essentially a disease of the sarcomere, characterized by reduced penetrance and highly variable expressivity. Although additional causative genes may be found, these will most likely explain only a minor proportion of cases, as demonstrated by the few non‐sarcomeric genes now deemed validated for HCM.

Recent findings and recommendations now point to a more focused application of genetic testing in HCM. Genes of unknown significance, whose association with the disease remains unproven, should be excluded from diagnostic testing as they offer a negligible increase in diagnostic yield but considerably increase the background noise of VUS.^25^, ^29^, ^33^, ^103^ Conversely, more comprehensive and focused analysis of known disease genes is likely to yield greater results than attempts to discover novel disease genes. This will include improved classification techniques for coding variants and detection of variant classes historically considered to be of low pathogenic potential, including intronic variants affect splicing, which recent research has highlighted are sometimes irrefutably pathogenic and could therefore contribute to resolving an additional proportion of unexplained disease cases.^81^, ^128^

A large proportion of HCM cases still remain without an identified causative variant, with 40% to 60% of screened individuals receiving an inconclusive or a negative test result.^49^, ^65^, ^102^, ^129^, ^130^ More complex genetic models for HCM are increasingly believed to hold the answer to the majority of these genetically elusive cases. These are still largely unexplored and could include a polygenic model, where multiple rare variants cause disease when carried by the same individual but are benign in isolation, and complex disease model combining the effects of common variants of low effect size with nongenetic factors such as hypertension and obesity. However, investigations into such models have so far been limited by the lack of availability of case and control cohorts large enough to reach satisfactory statistical power. To date, the only GWAS reported for HCM comprised 174 patients and 823 controls, and detected a significant association between a common intronic variant in the *FHOD3* gene on chromosome 18 and the presence of disease.^131^ Such efforts will be increasingly possible as single centers are more easily able to sequence large numbers of patients, and through publicly accessible (though with variable data access policies) phenotyped and sequenced population cohorts such as those by the National Institutes of Health (the ongoing “All of us” research program that aims at 1 million genomes, and the dbGap database^132^), the UK Biobank,^133^ Genomics England^134^ and the Cooperative Health Research in South Tyrol.^135^ Such data sets, coupled with data sharing efforts such as the SHaRe Registry, will plausibly enable investigations on multi‐genic and other complex disease models.

The lack of clear correlation between the genetic and clinical status of patients has until now dented hopes for the development of personalized medicine approaches, with genetic testing results still being used in a strictly diagnostic (rather than predictive) fashion. The incomplete penetrance of pathogenic sarcomeric variants, and the variable phenotypic expression in variant carriers, indicates that other genetic and non‐genetics factors are likely to influence the development of disease. It has been observed that the presence of multiple rare sarcomeric variants has a cumulative effect on disease onset and incidence of events, regardless of the variants’ diagnostic classification (ie, even when the variants could not be classified as disease‐causing),^136^ even though previous studies on multiple co‐occurring sarcomeric mutations in HCM likely overestimated the pathogenicity of the individual variants.^137^, ^138^ This suggests that variants that are not pathogenic in isolation may act to modify and exacerbate the disease phenotype in presence of a disease‐causing allele. Research into the full range of genetic factors that may interact with primary sarcomeric mutations to influence phenotype is at an early stage but is promising—such factors could include common, low frequency, and rare variants that add to the mutational burden, act as protective variants, or regulate the ratio of expression of mutated and wild‐type alleles.^139^ Identifying these factors through large‐scale genome‐wide association studies and WGS, and integrating such a genetic profile of common and rare variants with known nongenetic modifying factors such as hypertension and obesity, offers the promise in the near future of improved risk prediction for HCM patients and their relatives and a more personalized approach to clinical care.

## Conclusions

The path to a complete and detailed understanding of how genetic variants cause HCM and determine its severity will be long and challenging. Three major challenges remain unresolved and need to be addressed:

1. The still suboptimal accuracy of variant interpretation strategies,
2. The scarcity of clear genotype‐phenotype correlations, and
3. The necessity to expand research on genetic causes of HCM beyond the monogenic Mendelian inheritance model.

Advances in our understanding of population genetics, the development of new functional genomics techniques and quantitative approaches to variant interpretation, data sharing of cohort data and evidence from clinical genetics laboratories, and the introduction of new standards for assessing gene and variant pathogenicity have all greatly improved our understanding of the Mendelian genetics of HCM.

Genetic testing can now be more rigorously and consistently applied for HCM patients, enabling the accurate identification of primary causative variants and at‐risk individuals.

Our understanding of the more complex genetics that may underlie both the highly variable phenotypes observed in sarcomeric variant carriers and the genetic basis of currently genotype negative and likely non‐Mendelian HCM is still at an early stage. It is likely that significant investment in sequencing and phenotyping, and further initiatives in developing international registries and consortia, will be essential for allowing us to make similar progress in understanding the complex genetics underlying HCM.

## Sources of Funding

This work was supported by the Horizon 2020 Framework Programme (GA777204 – SILICOFCM); the Italian Ministry of Health (RF‐2013‐02356787); the Italian Ministry of Education, University and Research; the Cardiovascular Research Centre – Royal Brompton & Harefield NHS Trust and the NIHR Imperial College Biomedical Research Centre; and from Amsterdam Cardiovascular Sciences.

## Disclosures

None.

## Supporting information


**Table S1**
