# Supplementary material for: Contemporary Insights Into the Genetics of Hypertrophic Cardiomyopathy: Toward a New Era in Clinical Testing?
Source: J Am Heart Assoc. 2020 Apr 18;9(8):e015473. doi: 10.1161/JAHA.119.015473 (PMC7428545; doi:10.1161/JAHA.119.015473)
Supplement: Supplementary file 1 — Table S1 [file JAH3-9-e015473-s001.pdf]

SUPPLEMENTAL MATERIAL

Table S1. List of genes with ≥1 variant implicated in HCM in the Human Gene Mutation Database (HGMD) version 2016.3, alongside year of first published association with HCM and relative publication (PubMed ID), and gene classification by the ClinGen curation effort (Ingles et al, Circ Genom Precis Med, 2019 — PMID 30681346)

GENE	YEAR	PMID	CLINGEN CLASSIFICATION
ACTA1	2006	16945537	No evidence (isolated HCM)
ACTC1	1999	10330430	Definitive (isolated HCM)
ACTN2	2010	20022194	Moderate (intrinsic cardiomyopathy gene - isolated HCM)
ANKRD1	*2009*	19608031	Limited (isolated HCM)
BRAF	2012	22589294	NA
CACNA1C	2014	24183960	Definitive (Timothy syndrome - syndromic conditions where isolated HCM may be seen)
CALR3	2007	17655857	Limited (isolated HCM)
CASQ2	2007	17655857	No evidence (isolated HCM)
CAV3	2004	14672715	Definitive (Caveolinopathy - syndromic conditions where left-ventricular hypertrophy is seen only with overt systemic features)
COX15	2003	12474143	Strong (Leigh syndrome - syndromic conditions where left-ventricular hypertrophy is seen only with overt systemic features)
CRYAB	2013	23197161	Definitive (Alpha-B crystallinopathy - syndromic conditions where left-ventricular hypertrophy is seen only with overt systemic features)
CSRP3	2003	12642359	Moderate (isolated HCM)
DES	2006	16585054	Definitive (Desminopathy - syndromic conditions where isolated HCM may be seen)
FHL1	2012	22923418	Definitive (Emery-Dreifuss muscular dystrophy - syndromic conditions where isolated HCM may be seen)
FHL2	2014	25358972	NA
FXN	2005	15936968	Definitive (Friedrich ataxia - syndromic conditions where left-ventricular hypertrophy is seen only with overt systemic features)
GAA	2012	22555271	Definitive (Pompe disease - syndromic conditions where left-ventricular hypertrophy is seen only with overt systemic features)
GLA	2012	22336178	Definitive (Fabry disease - syndromic conditions where isolated HCM may be seen)
JPH2	2007	17509612	Moderate (isolated HCM)
KCNQ1	2014	24183960	No evidence (isolated HCM)
KLF10	2012	22234868	Limited (isolated HCM)
LAMP2	2011	21896538	Definitive (Danon disease - syndromic conditions where isolated HCM may be seen)
LDB3	2006	17097056	Definitive (Myofibrillar myopathy - syndromic conditions where left-ventricular hypertrophy is seen only with overt systemic features)
LMNA	2013	23785128	NA
MAP2K1	2012	22589294	NA
MAP2K2	2012	22589294	NA
MRPL3	2011	21786366	NA
MYBPC3	1990	1975599	Definitive (isolated HCM)
MYH6	2002	11815426	Limited (isolated HCM)
MYH7	1989	2811944	Definitive (isolated HCM)
MYL2	1996	8673105	Definitive (isolated HCM)
MYL3	1996	8673105	Definitive (isolated HCM)
MYLK2	2001	11733062	Limited (isolated HCM)

MYO6	2004	15060111	Definitive (Bilateral hearing loss - syndromic conditions where left-ventricular hypertrophy is seen only with overt systemic features)
MYOM1	2011	21256114	Limited (isolated HCM)
MYOZ2	2007	17347475	Limited (isolated HCM)
MYPN	2010	20801532	Limited (isolated HCM)
NEBL	2016	27186169	NA
NEXN	2010	20970104	Limited (isolated HCM)
OBSCN	2007	17716621	Limited (isolated HCM)
PDLIM3	2010	20801532	Limited (isolated HCM)
PKP2	2015	26332594	NA
PLN	2015	26573135	Definitive (intrinsic cardiomyopathy gene - isolated HCM)
PRKAG2	2001	11371514	Definitive (PRKAG2 cardiomyopathy - syndromic conditions where isolated HCM may be seen)
PTPN11	2012	22555271	Definitive (Noonan syndrome - syndromic conditions where isolated HCM may be seen)
RAF1	2007	17603483	Definitive (Noonan syndrome - syndromic conditions where isolated HCM may be seen)
RIT1	2014	24901346	Definitive (Noonan syndrome - syndromic conditions where isolated HCM may be seen)
RYR2	2015	26573135	Limited (isolated HCM)
SCN5A	2016	26656175	NA
SGCD	2007	17652892	NA
SLC25A4	2005	16155110	Definitive (Mitochondrial disease - syndromic conditions where left-ventricular hypertrophy is seen only with overt systemic features)
SOS1	2012	22555271	NA
SRI	2004	16118855	NA
TCAP	2004	15582318	Limited (isolated HCM)
TNNC1	2001	11371514	Moderate (isolated HCM)
TNNI3	*1997*	9241277	Definitive (isolated HCM)
TNNT2	1994	8205619	Definitive (isolated HCM)
TPM1	1994	8205619	Definitive (isolated HCM)
TRIM54	2014	24865491	NA
TRIM55	2014	24865491	NA
TRIM63	2012	22821932	Limited (isolated HCM)
TTN	1999	10462489	Limited (isolated HCM)
TTR	2015	25611685	Definitive (Transthyretin amyloidosis - syndromic conditions where isolated HCM may be seen)
VCL	2006	16712796	Limited (isolated HCM)
